# Study of Cat Allergy Using Controlled Methodology—A Review of the Literature and a Call to Action

**DOI:** 10.3389/falgy.2022.828091

**Published:** 2022-03-17

**Authors:** Lubnaa Hossenbaccus, Sophia Linton, Rashi Ramchandani, Alyssa G. Burrows, Anne K. Ellis

**Affiliations:** ^1^Department of Medicine, Queen's University, Kingston, ON, Canada; ^2^Allergy Research Unit, Kingston Health Sciences Centre – KGH Site, Kingston, ON, Canada; ^3^Department of Biomedical and Molecular Sciences, Queen's University, Kingston, ON, Canada

**Keywords:** cat allergies, allergen exposure chamber (AEC), nasal allergen challenge, natural exposure model, allergic rhinitis (AR), Fel d 1 exposure

## Abstract

The prevalence of cat allergen-induced AR is increasing worldwide, prompting its study using controlled methodology. Three general categories of allergen exposure models currently exist for the study of cat allergen-induced AR: natural exposure cat rooms, allergen exposure chambers (AEC), and nasal allergen challenges (NAC). We evaluated existing literature surrounding the use of these models to study cat allergen induced AR using online research databases, including OVID Medline, Embase, and Web of Science. We report that natural exposure cat rooms have been important in establishing the foundation for our understanding of cat allergen-induced AR. Major limitations, including variable allergen ranges and differing study designs highlight the need for a more standardized protocol. In comparison, AECs are an exceptional model to mimic real-world allergen exposure and study long-term implications of AR with large sample sizes. Existing AECs are limited by heterogeneous facility designs, differing methods of cat allergen distribution, and issues surrounding cost and accessibility. Conversely, NACs allow for smaller participant cohorts for easier biological sampling and are ideal for phase I, phase 2 or proof-of-concept studies. NACs generally have a standardized protocol and are less expensive compared to AECs. Nevertheless, NACs solely capture acute allergen exposure and have the further limitation of using allergen extracts rather than natural allergen. As the use of combined controlled methodologies is sparse, we recommend concurrent use of AECs and NACs to study short- and long-term effects of AR, thereby providing a more holistic representation of cat allergen-induced AR.

## Introduction

Pet dander is a major source of indoor allergen and a common cause of perennial allergic rhinitis (AR). The prevalence of cat allergen-induced AR specifically, caused by domestic cats (*Felis domesticus*), is increasing worldwide (1–5). There are 10 unique allergens that have been identified originating from cats found in the saliva, pelt, or urine, with Fel d 1 being the primary culprit of AR (6–8). In sensitized patients, exposure to cat allergens trigger an immunoglobulin E (IgE)-mediated inflammatory response in the nose, resulting in the characteristic symptoms of AR including sneezing, itchy nose, nasal pruritus, and congestion.

**Figure 1:**
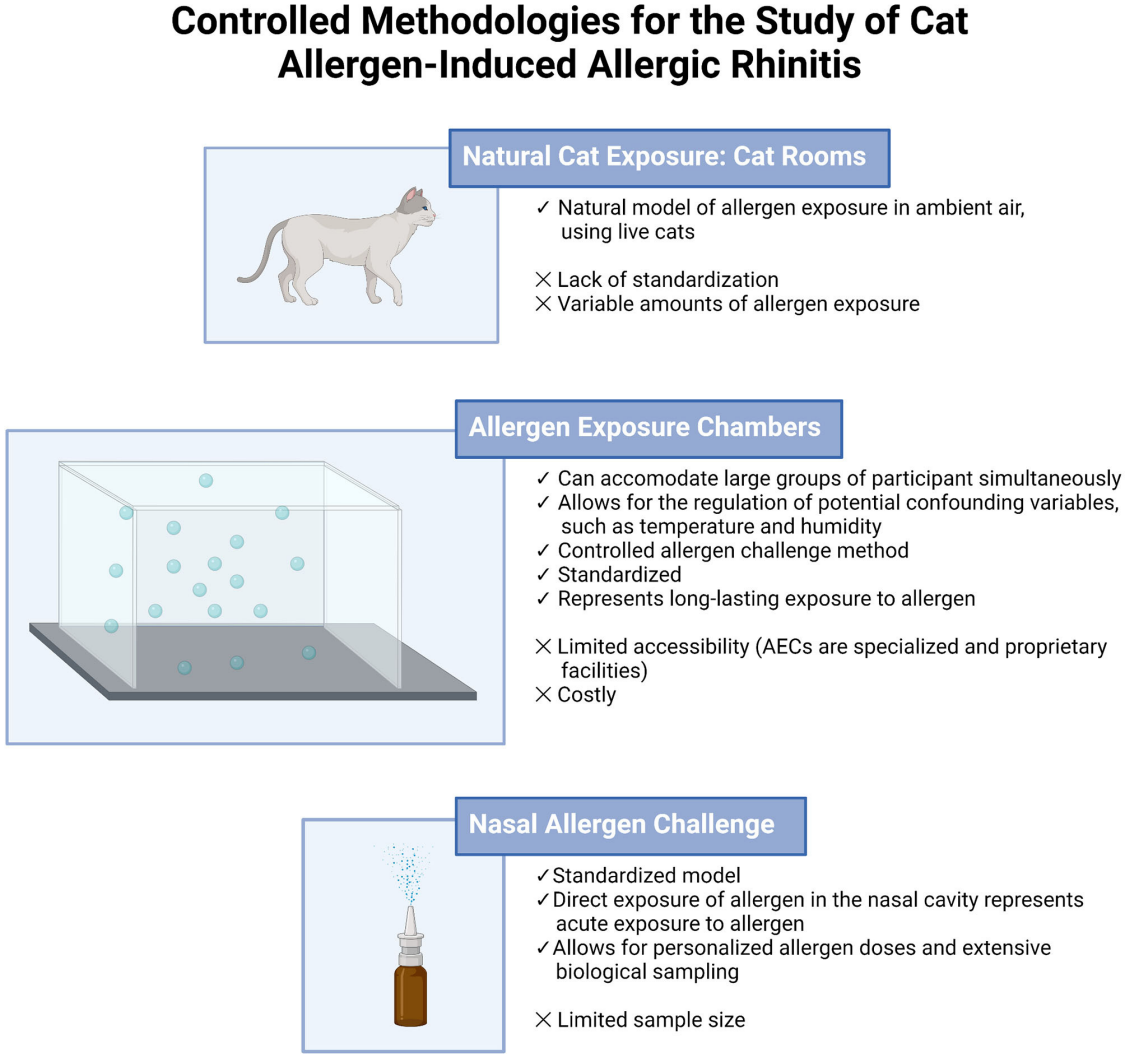
Comparison of models used in the investigation of cat allergy.

In the study of AR, controlled methodologies aim to regulate exposure of the target allergen while minimizing the effect of extraneous variables (Figure 1). Historically, natural exposures were conducted, where participants were placed in an environment where they were exposed to the allergen of interest for a certain period. For cat allergen-induced AR, cat rooms were used extensively. While allowing for natural exposure, such models involve a wide range of allergen concentrations, sometimes resulting in higher levels than are normally observed in a home setting (9, 10). In contrast, allergen exposure chambers (AEC) mimic natural exposure while controlling all environmental variables, allowing for more precise and controlled distribution of allergen. They are specialized facilities, requiring trained staff, and are limited in number (11). AECs are ideal for representing longer-lasting exposures to allergen, whereas the nasal allergen challenge (NAC) is an acute and localized model (12). NAC protocols may vary, though it offers the opportunity to provide personalized doses of allergen intranasally to participants and extensive biological sampling (13). AEC and NAC models are useful for investigating disease pathophysiology and for the evaluation of AR therapies.

In this review, we performed a literature search using online databases, including OVID Medline, Embase and Web of Science and have summarized the current state of the literature on studying cat allergen-induced AR using controlled clinical methodologies.

## Natural Cat Exposure: Cat Rooms

Cat rooms draw upon the natural mode of allergen exposure in ambient air (14). Generally, they are small, constructed rooms containing one to two neutered cats who live in the space for several days; sometimes a litter box is also kept in the room. Depending on the study design, the cats are either kept in their cages or are free to roam around when participants are in the room (15–21). Most studies include the vigorous shaking of a blanket at a certain time interval to disturb the allergen (15–18, 20–22). In a study by Berkowitz *et* al., their cat exposure room shared the same ventilation supply as a cat shelter containing 80 cats. To aerosolize the cat allergen, they vigorously shook the cats' bedding before participant entry and at 15-min intervals (22). Generally, Fel d 1 concentrations in cat rooms are measured using enzyme linked immunosorbent assays (ELISA) (Table 1). The natural cat exposure experimental model has been used to evaluate the pathophysiology of cat allergen-induced AR, pharmacotherapies, and sublingual (SLIT) and subcutaneous (SCIT) allergen-specific immunotherapy (AIT) options for patients.

**Table 1:** Summary of cat room studies.

**References**	**Date**	**Design**	**Outcome**	**Allergen level (Feld-d1 measured by ELISA ng/m^**3**^) unless reported otherwise**.	**Symptom reporting**	**Physiological measurements**
**Pathophysiology studies**						
Wanger et al. (23)	1999	Evaluation of SPT sensitivity to airway hyperresponsiveness (*n* = 29)	- All 29 subjects had a positive skin test (wheal ~ 4 mm), but only 12 (41%) had a positive airway response (fall in FEV, ~15%). - There were no significant correlations between wheal size and percent change in FEV_1_ (*r* = −0.19; *P*= 0.329) - Scores for all five symptoms increased significantly (i.e., worsened) from baseline.	170 to 1,260 ng/m^3^	5 Nasal and Ocular Symptoms	FEV_1_, SPT
Raizman et al. (20)	2000	- Two prospective, nonrandomized comparative studies, evaluating effect of eye rubbing on signs and symptoms of allergic conjunctivitis (*n* = 20)	- After eye rubbing without cat exposure, rubbed eye exhibited significantly increased itching and chemosis at 5 and 15 min and increased hyperemia at 5 min compared to non-rubbed eye. - Eye rubbed during cat room exposure exhibited significantly increased itching at 5, 15, 30 and 60 min, significantly increased chemosis at 5 and 15 min, and increased hyperemia at 5, 15, and 30 min when compared to the non-rubbed eye.	265.6 to 3214.2	Ocular itching	N/A
Zeidler et al. (24)	2006	Investigating the role of small airways in cat allergen induced reactions in people with mild asthma (*n* = 10).	- There was no significant decline in FEV_1_ at 6 or 23 h after cat exposure. - 25% and 75% forced vital capacity was reduced at 6 h post-challenge returning to normal at 23 h. - HRCT found significant increase in air trapping at both 6 and 23 h after the challenge - Significant increase in small airway hyperresponsiveness to methacholine was observed 23-h post-challenge.	117 ± 79.5	N/A	PFT, FEV_1_, HRCT
**Non-pharmacologic interventions**						
Wood et al. (25)	1998	Placebo controlled trial of HEPA Air Cleaner in people with cat induced asthma and rhinitis (*n* = 35)	- Fel d 1 levels were reduced in the active group's bedrooms were reduced compared to the placebo group's homes (*p* = 0.045) - No significant differences in nasal, chest symptoms or medication use were found.	Fel d 1 levels in bedrooms with HEPA filters changed from 3.0 ng/m^3^ to 3.2, 1.9, 1.7 ng/m^3^ at months 1, 2, 3, respectively. Placebo groups Fel d 1 levels changed from 2.6 ng/m^3^ to 2.4, 2.8, and 2.8 ng/m^3^ within the same time frame. HEPA filters were installed at month 2 in the active group.	Nasal symptom score, chest symptom score, medication use	PFR
**Pharmacologic and biologic studies**						
Corren et al. (19)	2001	Randomized, double-blind, placebo-controlled crossover design (*n* = 18) 20 mg zafirlukast twice daily for one week to eighteen	- Zafirlukast significantly improved the pre-challenge baseline FEV_1_ (P 0.001) and attenuated the decrease in FEV_1_ induced by cat challenge (P 0.019). - Zafirlukast also significantly reduced lower airway symptoms associated with cat challenge (*P* 0.005) but had no effects on nasal symptoms. - Active group had no significant differences when compared to the placebo group in regards to sputum inflammatory cells or eosinophil cationic protein, it significantly reduced the absolute counts of total white cells, lymphocytes, neutrophils, and basophils in nasal lavage fluid.	1,029 (treatment group), 981 (placebo group)	Upper and lower respiratory symptoms	FEV_1_, Sputum and Nasal Lavage samples (total cell count, cell differential, ECP)
Berkowitz et al. (22)	2006	Single-center, randomized, double-blind, placebo-controlled, 2-way crossover study (*n* = 63) Single dose of 180 mg fexofenadine hydrochloride given 90 min before allergen challenge	- Significant improvement in sneezing (0.004), and all other symptoms trended toward improvement in treatment group compared to placebo group. - Fexofenadine treated group showed smaller mean ± SD decreases in PEFR (−12.9 ± 5.26 L/min, −2.7% ± 1.29%) compared with placebo users (-27.6 ± 5.26 L/min; −6.0% ± 1.29%), with a statistically significant difference observed 30 min after allergen challenge between treatment groups (*P* = 0.03) - Fexofenadine treated group showed smaller non-significant decreases in FEV_1_ compared with placebo users.	2,646.1 ± 2,271.7 (treatment group), 2,700.7 ± 2,044.9 (placebo group)	TSS	PNIF, PEFR, FEV_1_
Corren et al. (15)	2011	Double-blind, placebo-controlled, parallel-group study 16 weeks of subcutaneous omalizumab vs. Placebo in patients with moderate asthma and history of cat allergen (*n* = 69).	- At 16 weeks, Omalizumab treated participants (*n* = 36) had a significantly lower percentage decrease of FEV_1_ at 20 min of challenge compared to placebo group (*n* = 33) - Omalizumab treated participants tolerated longer allergen exposure and demonstrated significant reduction from pre-challenge values in chest symptom score (*P* < 0.0001) and nasal-ocular symptom scores (*p* = 0.0002)	0 to 22,631	NOSS, chest symptom score	FEV_1_, Exhaled NO levels, SPT
**Immunotherapy**						
Nelson et al. (26)	1993	Double-blind, placebo controlled evaluation of SLIT (n=40)	- Participants treated with cat dander SLIT had fewer but non-significantly different symptoms and nasal congestion - No changes in IgG and IgE levels, or SPT reactions were found	69.5 ± 43.7 Allergen Units/8 h (Apartment)	HEENT, CNS, respiratory, musculoskeletal	SPT, cat specific sIgG, sIgE
Varney et al. (27)	1997	Randomized, double blind, placebo-controlled trial in people with moderate to severe allergic rhino conjunctivitis (*n* = 28)	- Treatment group had a significant reduction in symptoms during cat exposure (mean score 61.6–17.1, *P* < 0.001) whereas, the placebo group had no change from baseline (64.7 vs. 62.1) - Significant reduction of PEFR mean fall of 85 L/min pre-treatment, 29 L/min after treatment. P < 0.005 in active group, no significant change in placebo group. - Reduction in SPT sensitivity to cat and dust mite allergen extract, no change to histamine or codeine.	Carpet: 23, 212 ng Fel d l/g dust Chairs: 41, 006 ng/g (House)	Chest, nose, eyes, throat	PEFR, SPT
Álvarez-Cuesta et al. (28)	2007	Randomized double blind placebo controlled clinical trial of cat SLIT over 1 year (*n* = 50)	- The SLIT treated group had a 62% symptom reduction during natural challenge test which was significant compared to placebo (*P* < 0.001) - The active group experienced significant reductions in PEF response to cat allergen (*P* < 0.005) and improvement in skin reactivity (*P* < 0.005).	6.2 ± 2.21	TSS, Nasal, bronchial and ocular	PEF, SPT

*CNS, Central nervous system; HEENT, head, ears, eyes, nose and throat; HEPA, high-efficiency particulate air cleaner; HRCT, high-resolution computed tomography; ECP, eosinophil cationic protein; FEV_1_, Forced expiratory volume in the 1st second; NO, nitric oxide; NOSS, nasal ocular symptom score, PEFR; peak expiratory flow rate; PNIF, peak nasal inspiratory flow; PFT, pulmonary function testing; TSS, total symptom score*.

### Pathophysiology Studies

A handful of studies using cat rooms have investigated the physiological impact of cat allergen exposure in sensitized participants. Wagner et al. evaluated the correlation between skin testing reactivity and fall in forced expiratory volume in one second (FEV_1_) in cat sensitized individuals (23). They reported no significant correlations between the wheal size and percent change in FEV_1_ (*r* = −0.19; *P* = 0.329). All five symptom scores recorded (itchy/watery eyes, rhinorrhea, cough, nasal congestion) were worse compared to baseline after cat exposure (*P* < 0.001; *P* = 0.003). Zeilder et al. investigated the involvement of the small airways (<2 μm in diameter) in the late allergic asthmatic response induced by natural exposure to inhaled cat allergen (24). They reported that 20% of Fel d 1 particles were <6 μm while 7% were <3.2 μm. At 6 h following challenge, there was a borderline significant decline of FEV_1_ and a significant decline of forced expiratory flow from 25 to 75% of forced vital capacity at 6 h, which was undetectable at 23 h. High-resolution computed tomography found a significant increase in air trapping at both 6 and 23 h after the challenge. Additionally, a significant increase in small airway hyperresponsiveness to methacholine was observed 23-h post-challenge. These results demonstrate worsening of small airway obstruction at 6- and 23-h post-cat exposure.

Ocular symptoms have also been evaluated in participants with allergic conjunctivitis before, during, and after cat exposure (20). Eye rubbing during cat room exposure resulted in significantly increased itching, chemosis, and hyperemia compared to the non-rubbed eye. Prolonged redness and itchiness were also observed during cat exposure compared to eye rubbing without cat exposure. This study demonstrated that eye rubbing may play a role in ocular signs and symptoms in cat allergen allergic conjunctivitis and encouraging patients to stop rubbing their eyes may improve their clinical course.

### Pharmacologic and Biologic Studies

The primary approach to AR management, following allergen avoidance, is pharmacotherapy, characterized by antihistamines, corticosteroids, leukotriene receptor antagonists, and combination therapies. In this section, we explore pharmacotherapies and biological agents for AR evaluated using cat rooms.

Berkowitz et al. evaluated fexofenadine hydrochloride, an H_1_ antagonist, in a single-center, randomized, double-blind, placebo-controlled, 2-way crossover study (*n* = 63) using ventilated air from a cat shelter housing 80 cats (22). Participants were given a 180 mg dose of fexofenadine hydrochloride and after 90 min, were exposed to cat allergen in the cat room. At 30 min post-challenge, the treatment group also had a significantly less (*P* = 0.03) mean (± SD) decreases in peak expiratory flow rate (PEFR) (−12.9 ± 5.26 L/min, −2.7% ± 1.29%) compared to placebo (−27.6 ± 5.26 L/min; −6.0% ± 1.29%). At 60 min post-onset of cat allergen exposure, participants in the treatment group had significant improvements in sneezing (*p* = 0.004) and all other symptoms trended toward improvement compared to the placebo group. Only the placebo group experienced a significant mean (± SD) decrease in FEV_1_ of −0.041 ± 0.015 L/s (*P* = 0.007) compared with their pre-challenge baseline values, suggesting a protective effect for antihistamine, fexofenadine.

In a randomized, double-blind, placebo-controlled, cross-over study, zafirlukast, an oral leukotriene receptor antagonist, was evaluated (19). Participants (*n* = 18) were given 20 mg zafirlukast twice daily for one week before an allergen challenge. After cat exposure, participants who had received zafirlukast had significantly attenuated decrease in FEV_1_ compared with placebo (−15.1 ± 2.7 vs. −25.1 ± 2.7, *P* = 0.019). Symptom scores decreased by 38% in the treatment group compared to the placebo; both wheezing (*P* = 0.004) and chest tightness (*P* = 0.0019) were significantly reduced, although it is worthwhile to recognize that a validated AR symptom scoring system was not used. Nasal lavage fluid revealed significantly fewer total cells and absolute counts of lymphocytes, neutrophils, monocytes, and basophils but no significant differences in the absolute or percentage values of eosinophils or eosinophil cationic proteins (ECP). Overall, this study demonstrated the effectiveness of zafirlukast at reducing acute pulmonary responses to cat exposure, though a longer duration of treatment may be needed to evaluate biological changes.

A newer treatment approach for AR involves omalizumab, an anti-IgE monoclonal antibody that is widely used to treat allergic asthma through the inhibition of IgE binding to the FcER1 receptor on mast cells and basophils. In a natural cat exposure study, omalizumab was administered at a dose of 0.008 mg/kg/IgE [IU/mL] every 2 weeks or 0.016 mg/kg/IgE [IU/mL] every 4 weeks (based on body weight and baseline serum total IgE levels) for 16 weeks (15). Some participants had cats in their homes whereas others did not and were randomized accordingly. Omalizumab-treated participants had a 44% improvement in the area under the curve (AUC) of the percentage decrease from pre-challenge FEV_1_ during a 60-min cat chamber exposure compared to the placebo-treated patients (15.2% per hour vs. 27.3% per hour, *P* = 0.0009). At 16 weeks, significant reductions in chest symptom scores (*P* < 0.0001), nasal ocular symptom scores (NOSS) (*P* = 0.0002), and skin prick reactivity (*P* < 0.0001) were observed in the treatment group. Omalizumab-treated participants tolerated a median of 50 min in the cat room whereas the placebo group tolerated a median of 22 min (*p* = 0.0006). Across the three sites that this study was conducted, the Fel d 1 allergen varied greatly, specifically site 3 (Mississauga, ON), which had large variations and higher concentrations when compared to sites 1 and 2 and between their placebo (178 to 6,445 ng/m^3^) and treatment group (19 to 4,744 ng/m^3^). Omalizumab was shown to significantly reduce symptom scores and improve FEV_1_ upon allergen exposure after 16 weeks of treatment however, results should be interpreted cautiously given the inconsistencies in allergen exposure.

### Immunotherapy Studies

SLIT and SCIT options for cat allergen-induced AR have been evaluated using the cat room model, with Fel d 1 being commonly used in the AIT protocols.

A SCIT regimen of 100 units every two weeks to a maintenance dose of 100,000 units (15 μg of Fel d 1) given every four weeks was evaluated in a double-blind placebo-controlled trial in 28 patients by Varney et al. The exact duration of treatment is unclear. All participants were brought to a house where three cats had lived for over 8 years and Fel d 1 concentrations were measured from carpets and chairs (Table 1). The SCIT treatment group had a significant reduction in symptoms during cat exposure (*P* < 0.001) whereas, the placebo group had no change when compared to baseline. A significant reduction in decreased PEFR (*P* < 0.005) was found only in the active group, with a reduction in SPT sensitivity to cat extract. Together these results suggest that cat-specific SCIT therapy successfully reduced symptoms, PEFR, and SPT reactivity as evaluated in a natural exposure model (27).

Nelson et al. conducted a 105-day double-blind, placebo-controlled evaluation of SLIT therapy to 4,500,000 allergy units of cat extract in 40 patients with AR with and without asthma after cat exposure (26). At the end of the course of treatment, participants were brought to an apartment that had cat dander for a 90-min exposure. Following exposure, participants treated with cat dander SLIT had fewer but non-significantly different symptoms and nasal congestion. Additionally, no changes in IgG and IgE levels or skin prick testing reactions were observed. This study was critiqued by Bousquet et al. in a letter to the editor, specifically that the SLIT treatment duration was likely not long enough to make a definitive conclusion about the treatment efficacy (29).

Following this, Alvarez-Cuesta et al., conducted a double-blind, placebo-controlled study to evaluate the effectiveness of SLIT on 50 patients over one year. During the SLIT build-up phase, the accumulated dose was 1.7 μg of Fel d 1. The total accumulated dose during the entire length of the study was 17.1 μg of Fel d 1, patients were instructed to keep the SLIT mixture under their tongue for 2 min prior to swallowing. Patients were advised to avoid cat exposure. After one year of treatment, the SLIT treated group had a 62% symptom reduction during natural challenge test (i.e. cat room), which was significant compared to placebo (*P* < 0.001). The active group also experienced significant reductions in PEF response to cat allergen (*P* < 0.005) and improvement in skin reactivity (*P* < 0.005) (28).

### Non-pharmacological Interventions

A non-pharmacological high-efficiency particulate air (HEPA) filter intervention was investigated using a natural exposure model in 35 cat-sensitized people with asthma and rhinitis who owned one or more cats (25). Fel d 1 concentrations were measured in participant's bedrooms at baseline, 1, 2, and 3 months. Participants were randomized and blinded to either receive an active HEPA filters or a non-active filter. Although a significant reduction in Fel d 1 concentrations in bedrooms was found between the active and non-active groups at months 2 and 3 (*p* = 0.045), the reduction in allergen levels in the bedroom was insufficient at decreasing medication use, and chest and nasal symptom scores. HEPA filters may be valuable in reducing allergen levels, however allergen levels outside the bedroom likely impact patients' disease response.

### Limitations

Exposure to cat allergen through cat rooms was sufficient to induce AR symptoms in cat-sensitized individuals with and without asthma and those with rhinoconjunctivitis. This model adequately assessed the benefits of pharmacological therapies, immunotherapies, and physiological changes due to cat exposure. However, the lack of standardization in the design and execution of cat rooms is of concern. Cat rooms have varying amounts of allergen exposure between room designs and the range of allergen within the same room throughout the experiment is variable. In the cat room studies, various symptom questionnaires and biological measurements were employed, making it difficult to compare results between studies (Table 1). While natural cat allergen exposure room having produced useful insights on cat allergen-induced AR, there is a need to standardize the design and execution of cat allergen exposure with a more robust clinical model.

## Allergen Exposure Chambers

AECs are controlled clinical models of AR that allow for a large participant population to be simultaneously exposed to a specified allergen concentration, with the regulation of all variables including, but not limited to, temperature and humidity (30). Keeping the current COVID-19 pandemic in mind, modifications to the protocol can be implemented to ensure safety of all participants and staff involved, such as ensuring all participants are double vaccinated, a negative PCR test result from 48 to 72 h prior to the study visit, negative rapid antigen testing upon arrival at the study site, and masking until the allergen challenge starts. While controlled, AECs in many ways simulate real-world exposure to cat allergen, especially as the allergen is usually aerosolized and moves in particle clusters with a diameter of <5 microns (8). A comparative study by Haya et al. found that in naturalistic environments, blanket shaking produced larger dander particles (2–40 μm), while expulsions from vacuum cleaners produced smaller sized particles (1–20 μm) (31). In AECs, the spread of allergen is enhanced by the presence of fans; this model provides an unique method of simulating real-world cat allergen exposure (32, 33).

AECs have been used to study participants' biological responses to cat allergen-induced AR. Sicherer et al. found that exposure to cat allergen through an AEC reduced participants FEV_1_, which is reflective of typical allergen bronchoprovocation (34). Interestingly, the bronchoprovocation responses observed in the AEC were used to identify patients with asthma, suggesting its usefulness in studying a spectrum of allergic manifestations associated with cat allergens. A validation study by Marcelo et al. found that levels of cat dander on the walls and floors of an exposure chamber were comparable to levels seen in residences (21). This highlights the usefulness of AECs, in that the levels of cat allergen in the controlled facility are shown to be akin to real-world exposures.

### AR Therapies

AECs have also been used to evaluate AR therapies, specifically AIT peptides. Patel et al. used an environmental exposure chamber in a double- blind placebo design to measure the efficacy and safety of Fel d 1-derived peptide antigen desensitization (Cat-PAD) 1-year after the start of treatment. Nasal and ocular symptoms were effectively measured to ultimately provide evidence for the novel treatment (35). Similar outcomes were observed in a study by Couroux et al. where anti-synthetic peptide immuno-regulatory epitopes were tested. The AECs employed in each of the studies provided an effective method to measure a variety biological symptom data for AR, rhinoconjunctivitis and asthma manifestations (36). Furthermore, Hafner et al. tested CAT-PAD 4-weeks apart measuring participants TNSS score in 30-min intervals. Relative to the placebo, CAT-PAD decreased participants TNSS during the treatment period and two-years post-treatment (37). Notably, cat allergen peptide immunotherapy studies using AECs were also able to deduce the mechanisms associated with immunotherapy. For instance, a study by Worm et al. found that Fel d 1 can overlap with several MHC binding regions. This sheds light on binding affinity of developed Fel d 1 peptides and subsequently assisted in determining appropriate dosing for allergen immunotherapy (38). Mechanism-related data in a non-AEC study using T cell peptides for Fel d 1 by Oldfield et al. revealed that specifically post-immunotherapy, cat allergen sensitized participants exhibited decreased levels of pro-inflammatory interleukin levels, notably interleukin (IL)-4 and IL-13 relative to the placebo (39). The AEC model allows for a vast array of investigations in a regulated experimental setting.

### Limitations

The outcomes of AEC studies have not been directly compared to cat rooms. They similarly involve aerosolizing cat allergen to be spread across a large space, however in cat rooms, the allergen is directly from the animal source, whereas in AECs, the cat allergen is generally purified. While AECs are more controlled, much validation work is required to ensure effective allergen distribution and the maintenance of consistent protocols across various AECs. As they are specialized and proprietary facilities, AECs are limited in number worldwide and are costly. This highlights the need for a more accessible and cost-effective model in the study of cat allergies.

## Nasal Allergen Challenge

### Biological Outcomes After an NAC With Cat Allergen

NAC and their various protocols have been extensively studied in the context of seasonal allergens and house dust mite, though there is limited data in the literature concerning the effects of cat allergen exposure. Recently, a couple of robust NAC studies have been published which detail biological outcomes after a cat allergen NAC.

Scadding et al. used a previously validated grass pollen NAC protocol to study the effects of cat allergen NAC and saw local and systemic Th2-driven inflammatory responses among their participants (40). Cat allergen dilutions were administered in a titrated fashion ranging 500 to 10,000 bioequivalent allergen unit (BAU)/ml, until the participants reached a TNSS score of 8. Each participant received one 100 μl spray to each nostril at 10-min intervals. Post-NAC, they saw a dose-response in symptoms and elevated levels of nasal fluid tryptase at 5 min after challenge. Levels of eotaxin, IL-4,−5,−9, and−13 were also increased at 8 h. Surface expression of CD63 and CD107a was evaluated to measure peripheral basophil activation and were found to be greater at 6 h than at baseline, both in the presence and absence of *in vitro* allergen stimulation.

The standardized NAC protocol developed by the Allergic Rhinitis–Clinical Investigator Collaborative project (AR-CIC), part of the Allergy, Genes and the Environment Networks for Centres of Excellence resembles the protocol developed by Scadding et al. The AR-CIC protocol differs slightly in that a titration challenge (doses ranging from 4.9 to 5,000 BAU/ml) is performed one week before administration of a single-dose NAC (41). The allergen dose administered was either a cumulative dose (from the lowest to the qualifying concentration dose) or a qualifying concentration dose (100 μl of the qualifying concentration). Both cohorts saw neutrophils, increased at 1 and 2 h after NAC; eosinophils, decreased at 1 and 2 h after NAC; lymphocytes, increased at 6 h after NAC; and the neutrophil/lymphocyte ratio, increased at 1 and 2 h after NAC. This group also identified seven clusters of immune gene expression patterns after NAC and some clusters were associated with clinical symptoms or immune cell frequencies. These findings suggest there may be systemic immune response signatures in whole peripheral blood post-NAC.

Doherty et al. studied Type 2 innate lymphoid cells (ILC2s) using the NAC model with cat allergen (42). A titrated-NAC was conducted using increasing concentrations of cat allergen extract (4, 40, 400 BAU/ml) administered 10-min apart. Post-NAC, they saw an increased percentage of peripheral blood ILC2s that express the chemoattractant receptor homologous molecule expressed on Th2 lymphocytes (CRTH2). However, the role of increased peripheral blood ILC2s after NAC remains unclear.

Paterniti et al. compared the magnitude of cat allergen-induced basophil histamine release (BHR) to NAC and other biological outcomes (43). They also administered a titration challenge at 10-min intervals with doses of 10, 100, and 1,000 BAU/ml in participants with cat allergy. A positive NAC was defined as 5 total sneezes. They saw that a positive cat allergen-induced BHR is associated with higher cat-specific IgE levels, a higher cat-specific to total IgE ratio and is predictive of a positive cat-induced NAC. Sánchez et al. recently evaluated levels of IgE sensitization and symptom production in atopic and non-atopic participants using NACs with pet allergen extracts (44). They reported that significantly more allergic participants were sensitized to one or more cat allergen components compared to the control group (*p* = 0.05), with Fel d 1-specific IgE concentrations, in particular, being higher. IgE sensitization was not associated with a positive NAC outcome, however, those with higher IgE concentrations had increased probability of a positive challenge. These findings reinforce the need for better tools to effectively predict clinical outcomes to pet allergens.

Lastly, the NAC model has been used to compare the clinical presentation of cat allergen-induced AR with other allergens. In 2017, Steacy et al. compared the symptoms produced by ragweed and cat-NACs using the AR-CIC methodology (45). They saw that cat-allergic participants required a significantly higher dose to achieve the qualifying criteria of TNSS ≥ 8 and a %PNIF fall ≥60% during screening compared to ragweed-allergic participants. Both groups of allergic participants had the same peak TNSS post-challenge, however, the peak %PNIF fall was achieved at 15 min for cat-allergic participants and 30-min post-challenge. An important caveat to this study was that cat-allergic participants were required to live with a cat to participate in this study and ragweed-allergic participants were challenged out of season. This study suggests clinical differences in the presentation of AR against seasonal and perennial allergens, which may be useful to inform treatment practices.

### The Use of Cat-Allergen NACs in Clinical Trials

The biological outcomes of an NAC with cat allergen, described above, can be used as surrogate outcome measures in clinical trials. In the past 10 years, several trials investigating new pharmacotherapies and immunotherapy options for cat-allergy have been performed using the NAC as a model for cat-allergen exposure. These data build on previous research using NACs to evaluate onset of action and duration of efficacy for many therapeutics. Previously, Ewbank et al. performed a double-blind, placebo-controlled immunotherapy dose-response study with standardized cat extract and used a titrated NAC with cat hair extract to evaluate biological responses to treatment (46). A similar study by Nanda et al. assessed the long-term immunological response to reaching maintenance in immunotherapy (47). They also used a titrated NAC with cat hair and dander extract and saw the symptoms, nasal cytokines, and serum TGF-β levels at 5 weeks is predictive of the response at 1 year. These studies demonstrate the use of NAC in cat immunotherapy trials.

The effect of omalizumab on basophil and mast cell responses were evaluated using a cat NAC. This double-blind, placebo-controlled trial involved a baseline, mid-study, and final NAC with blood sampling to measure basophil histamine release (BHR) and basophil IgE/FcεRI measurements. The dosage of omalizumab was 0.016 mg/kg/IgE every 4 weeks. Eckman et al. saw a significant mean reduction in BHR and in mean combined nasal symptom scores by mid-study NAC (~4 weeks) compared to baseline suggesting basophils may play a role in the acute NAC response (48).

The use of Cat-PAD, a new form of immunotherapy for cat allergy, has been investigated using a cat NAC. In 2018, Neighbour et al. aimed to outline biomarkers of efficacy for Cat-PAF using AR-CIC NAC methodology (49). Participants underwent a baseline titrated NAC, followed by four intradermal injections of 6 nmol of Cat-PAD every 4-weeks and a final NAC. They saw a significant reduction in TNSS and PNIF after treatment. In 2020, Kim et al. used the same methodology to measure the clinical response to Cat-PAD at the same dosing (50). Post-NAC blood differential cell counts, transcriptomic profiles, and symptoms were compared before and after treatment. Total nasal symptom score (TNSS) post-NAC was significantly reduced after treatment as well as frequencies of neutrophils, lymphocytes, and monocytes. There was also a significant neutrophil to lymphocyte ratio (NLR) reduction at baseline, and 1 and 2 h-post NAC, after Cat-PAD treatment. IL-1β had a significantly lower RNA expression after treatment and IFN-y had significantly higher RNA levels post-NAC after treatment. This group also saw changes in clustered gene expression patterns after treatment: integrin subunit alpha E *(ITGAE, CD103), CD180 (LY64)*, neural cell adhesion molecule 1 *(NCAM1, CD56)*, C-C motif chemokine receptor 7 (*CCR7, CD197*), and leucine-rich repeat neuronal 3. These findings suggest that peripheral blood biomarkers could serve as predictors of treatment efficacy.

Recently, a phase 1b, randomized, double-blind, placebo-controlled proof-of-mechanism study was conducted to evaluate the therapeutic potential of monoclonal IgG antibodies (REGN1908-1909), administered subcutaneously, for cat allergy. Orengo et al. used an NAC with cat hair extract to measure the TNSS AUC from pre-treatment challenge to day 8 challenge (51). They saw TNSS AUC from baseline to day 8 NAC significantly decrease in patients receiving a single dose of REGN1908-1909 (600 mg) compared to placebo. They also saw a clinically meaningful reduction in TNSS AUC up to 85 days after the first dose. Finally, REGN1908-1909 was well-tolerated by participants (52). Another group, Shamji et al., also performed at phase 1b study of REGN1908-1909 using an NAC with cat hair extract followed by extensive biological sampling. NAC was conducted on Study Days 8, 29, 57, and 85 using a titration protocol. Following treatment, significantly decreased levels of IL-4, IL-5, IL-13, TARC, and RANTES were observed in nasal fluid samples post-NAC (Day 8). Likewise, they saw inhibitory activity in the treatment group, measured as cat allergen-IgE complexes bound to B cells. Together, these data demonstrate proof of principle that a single dose of REGN1908-1909 can reduce symptoms in patients with cat allergy.

## Comparison of Controlled Methodologies for Cat Allergen-Induced AR

As cat rooms are not validated and standardized, they are challenging to compare between studies and with AEC and NAC studies. To our knowledge, the first comparison of cat allergen NACs and a cat exposure room was in 1997 by Sicherer et al. (34). They conducted an NAC using filter paper disks containing up to 50 μl of allergen at the following concentrations: 50, 500, and 5000 BAU/ml until a FEV_1_ fall of 20% was reached. Participants also sat for 1-h in a cat-exposure room for an environmental allergen challenge (EAC), which contained 2 female cats, where Fel d 1 levels ranged from 289 to 9,349 ng/m^3^ per session. The challenges were separated at least 1 week. They saw that nasal symptom scores during the EAC were correlated with those post-NAC. However, this study is limited by a small sample size (*n* = 13) and the large variation in Fel d 1 exposure levels in the EAC sessions.

The clinical and biological comparability of the NAC and AEC has also not been investigated thoroughly, especially with cat allergens. In 2019, Tenn et al. compared clinical outcomes from EEU and NAC studies using ragweed and reported the induction of symptoms of similar severity between the models, though with differing temporal patterns (53). More recently, in 2020, Larson et al. conducted a randomized trial comparing the NAC and environmental exposure chamber (EEC) (54). Participants received either an NAC followed by a 2-day challenge in an EEC (Group A) or a 2-day challenge in an EEC followed by NAC (Group B). A washout period of 28-days separated each challenge type. In the NAC, a single dose of 0.87 μg total Fel d1 was administered whereas the EEC phase lasted 3 h with an exposure of 10 to 500 ng/m3 Fel d 1 allergen. They saw that symptoms with NAC peaked earlier (15-min and 30-min) than EEC (>1 h) and had overall reduced magnitude of response. They also observed strong NAC and EEC correlations when the allergen exposure-induced changes in IL-5 and IL-13 when expressed as 2- to 8-h AUCs. Similarly, differentially expressed genes showed a similar magnitude of change with each challenge and the changes were highly correlated. Together, these data suggest that clinical outcomes of the NAC and EEC are temporally different but induce similar immunologic responses. However, this study is limited by sample size, the lack of a non-allergic control group, and that the total dose administered in the two challenges differed significantly.

## Unmet Needs in the Study of Cat Allergies

AECs and NACs represent the future of research on cat allergen-induced AR as standardized and reproducible controlled methodologies. AECs generally model allergen exposure over the course of hours whereas NACs, through a sudden burst of allergen extract into the nose, are more representative of acute exposure. In everyday life, cat allergen exposure can variably be short-term, such as on public transportation, or long-term, as in one's home environment if a cat is kept as a pet (55). Both circumstances may cause the triggering of AR symptoms in sensitized individuals, hence, for the complete understanding of cat allergen-induced AR, both modes of exposure need to be considered. As explored in the previous section, only a few studies evaluate the two through the AEC and NAC, however with certain limitations, so there exists the need for better comparisons between the models to improve our understanding of the pathophysiology of cat allergen-induced AR, with larger sample sizes and standardized protocols.

## Conclusions

In this review, we discuss three models to study cat allergies including natural exposure cat rooms, AECs, and NACs. In comparison to cat rooms, the use of AECs and NACs to investigate cat allergen-induced AR are limited. While these models have been extensively used in pharmacological studies of AR, there is a paucity of data investigating the biological outcomes of cat allergies using an AEC or NAC. Existing studies are limited by their small sample size, lack of controls, non-standardized protocol, and specified dosing methodology. Given the rising incidence, it is pivotal to develop an appropriate and controlled study model to study cat allergen-induced AR. Based on the review of existing literature, the consideration of which model to employ when studying cat allergen-induced AR depends on study objectives, cost, and resource accessibility. Given their similarity to real-world exposure, we recommend using a combination of AEC and NAC to evaluate participants AR response at different timepoints. This would provide valuable information about the acute and long-term effects of allergen exposure to better understand the full spectrum of cat allergen-induced AR.

## Author Contributions

LH, SL, RR, and AB wrote and edited the manuscript. AE ensured critical revision of the manuscript. All authors read and approved the final manuscript.

## Conflict of Interest

AE has participated in advisory boards for Abbvie, ALK Abello, AstraZeneca, Aralez, Bausch Health, Circassia Ltd., GSK, LEO Pharma, Merck, Novartis, Pfizer; has been a speaker for ALK Abello, Aralez, AstraZeneca, CSL Behring, Medexus, Novartis, Mylan, Pfizer, Sanofi and Takeda. Her institution has received research grants from ALK Abello, Aralez, AstraZeneca, Bayer LLC, Circassia, Green Cross, Merck, Medexus, Pfizer, Novartis, Sanofi, and Regeneron. She has also served as an independent consultant to Bayer LLC and Regeneron. The remaining authors declare that the research was conducted in the absence of any commercial or financial relationships that could be construed as a potential conflict of interest.

## Publisher's Note

All claims expressed in this article are solely those of the authors and do not necessarily represent those of their affiliated organizations, or those of the publisher, the editors and the reviewers. Any product that may be evaluated in this article, or claim that may be made by its manufacturer, is not guaranteed or endorsed by the publisher.

## Abbreviations

AEC: allergen exposure chambers
AIT: Allergen Specific Immunotherapy
AR: allergic rhinitis
AR-CIC: Allergic Rhinitis–Clinical Investigator Collaborative project
AUC: area under the curve
BHR: basophil histamine release
Cat-PAD: Cat-peptide antigen desensitization
CRTH2: chemoattractant receptor homologous molecule expressed on Th2 lymphocytes
EAC: environmental allergen challenge
ECP: eosinophil cationic proteins
EEC: Environmental Allergen Chamber
ELISA: Enzyme-Linked Immunosorbent Assay
FDA: Food & Drug Administration
FEV_1_: Forced expiratory volume in the 1st second
HEPA: high-efficiency particulate air
IgE: immunoglobulin E
IL: interleukin
ILC2s: Type 2 innate lymphoid cells
NAC: nasal allergen challenge
NOSS: nasal ocular symptom scores
PEFR: peak expiratory flow rate
SCIT: Subcutaneous Immunotherapy
SLIT: Sublingual Immunotherapy
TNSS: total nasal symptom score.
